# Impact of Anti-Retroviral Treatment and Cotrimoxazole Prophylaxis on Helminth Infections in HIV-Infected Patients in Lambaréné, Gabon

**DOI:** 10.1371/journal.pntd.0003769

**Published:** 2015-05-20

**Authors:** Saskia Janssen, Sabine Hermans, Martijn Knap, Alma Moekotte, Elie G. Rossatanga, Akim A. Adegnika, Sabine Bélard, Thomas Hänscheid, Martin P. Grobusch

**Affiliations:** 1 Center for Tropical Medicine and Travel Medicine, Department of Infectious Diseases, Division of Internal Medicine, Academic Medical Center, University of Amsterdam, Amsterdam, The Netherlands; 2 Centre de Traitement Ambulatoire (CTA), Lambaréné, Gabon; 3 Centre de Recherches Médicales de Lambaréné (CERMEL), Lambaréné, Gabon; 4 Institute of Tropical Medicine, University of Tübingen, Tübingen, Germany; 5 Clinical Infectious Diseases Research Initiative, Institute for Infectious Diseases and Molecular Medicine, University of Cape Town, Cape Town, South Africa; 6 Desmond Tutu HIV Centre, Institute for Infectious Diseases and Molecular Medicine, University of Cape Town, Cape Town, South Africa; 7 Department of Global Health, Amsterdam Institute for Global Health and Development, Academic Medical Center, University of Amsterdam, Amsterdam, The Netherlands; 8 Department of Internal Medicine, School of Medicine, Makerere University College of Health Sciences, Kampala, Uganda; 9 Department of Pediatric Pneumology and Immunology, Charité-Universitätsmedizin, Berlin, Germany; 10 Instituto de Microbiologia, Faculdade de Medicina de Lisboa, Lisbon, Portugal; University of Washington, UNITED STATES

## Abstract

**Background:**

Foci of the HIV epidemic and helminthic infections largely overlap geographically. Treatment options for helminth infections are limited, and there is a paucity of drug-development research in this area. Limited evidence suggests that antiretroviral therapy (ART) reduces prevalence of helminth infections in HIV-infected individuals. We investigated whether ART exposure and cotrimoxazole preventive therapy (CTX-P) is associated with a reduced prevalence of helminth infections.

**Methodology and Principal Findings:**

This cross-sectional study was conducted at a primary HIV-clinic in Lambaréné, Gabon. HIV-infected adults who were ART-naïve or exposed to ART for at least 3 months submitted one blood sample and stool and urine samples on 3 consecutive days. Outcome was helminth infection with intestinal helminths, *Schistosoma haematobium*, *Loa loa* or *Mansonella perstans*. Multivariable logistic regression was used to assess associations between ART or CTX-P and helminth infection. In total, 408 patients were enrolled. Helminth infection was common (77/252 [30.5%]). Filarial infections were most prevalent (55/310 [17.7%]), followed by infection with intestinal helminths (35/296 [11.8%]) and *S*. *haematobium* (19/323 [5.9%]). Patients on CTX-P had a reduced risk of *Loa loa* microfilaremia (adjusted odds ratio (aOR) 0.47, 95% CI 0.23-0.97, P = 0.04), also in the subgroup of patients on ART (aOR 0.36, 95% CI 0.13-0.96, P = 0.04). There was no effect of ART exposure on helminth infection prevalence.

**Conclusions/Significance:**

CTX-P use was associated with a decreased risk of *Loa loa* infection, suggesting an anthelminthic effect of antifolate drugs. No relation between ART use and helminth infections was established.

## Author Summary

The geographical distribution of helminth infections, which are highly prevalent in many areas, overlaps considerably with regions of high HIV sero-prevalence. The highest burden of infection is found in resource-poor settings, making it unattractive for the pharmaceutical industry to invest. Limited available treatment options and drug-resistance are increasing problems for soil-transmitted helminths, whereas for some other helminth infections, such as for the blood-dwelling microfilariae, effective and safe treatment options are still far from being optimal. Limited evidence suggests antihelminthic effects of antiretroviral therapy (ART) in HIV-infected individuals. We aimed to investigate whether ART or cotrimoxazole preventive treatment (CTX-P) reduces prevalence of helminth infection in HIV-infected individuals attending a primary HIV clinic in a semi-rural area in Gabon. The most important finding of our study was that the use of CTX-P was associated with a reduced prevalence of *Loa loa* microfilaremia. ART use was not associated with a reduced prevalence of helminth infections. Additional studies are needed to assess the effects of CTX on helminth infections, as this might be a promising safe and effective drug adding to the limited repertoire of anthelminthic drugs.

## Introduction

Globally, more than 2 billion people are estimated to be infected with soil-transmitted helminths, and the geographical distribution of these infections overlaps considerably with regions of high HIV sero-prevalence [1,2]. Helminth infections have been hypothesized to be factors driving the HIV-epidemic in Africa [3,4], which may be due to their effects on the host immune system, as demonstrated by an increased susceptibility to HIV infection and progression to AIDS [3]. However, the immunological interaction between the two infections is complex, and others have found results that are conflicting with this hypothesis [5].

Although treatment of intestinal helminth infections and schistosomiasis is relatively simple and cheap, current options are limited to a few drugs, and emergence of resistance is anticipated [6,7]. Currently, no really effective and safe drug is available for the treatment of filariases. Diethylcarbamazine (DEC) is only moderately effective and has to be administered under supervision, due to its toxicity. DEC or ivermectin treatment may cause serious adverse reactions due to microfilarial disintegration triggering a cytokine release [8]. This underscores the need for new drugs for the treatment of helminth infections.

Data on helminth co-infections in patients receiving ART is scarce, as well as information on possible ART effects on helminth infections. Studies investigated patients on ART and compared them to non-treated or HIV-negative controls, usually looking at all intestinal parasites [9–13]. Although the common theme is that parasite infections are reduced in patients on ART, the underlying mechanisms remain unclear. Naturally, improved cellular immunity is often mentioned to explain these findings [9,10], especially for protozoal infections such as cryptosporidiosis. However, several authors speculate on the contribution of drug effects, both from ART as well as from cotrimoxazole preventive therapy (CTX-P), on the reduction of parasite burden [9,11,13].

Whilst most studies were cross-sectional or used historic controls, one Ethiopian study in patients with newly diagnosed tuberculosis found a significant reduction in helminth infections over time only in HIV-positive as compared to HIV-negative individuals [11]. Authors speculate on the possible effects of ART or CTX-P to explain these findings. A cross-sectional study in Rwanda found a reduced risk of *T*. *trichiura* infection in patients using stavudine in combination with lamivudine and nevirapine (OR, 0.27; 95% CIs, 0.10–0.76; p<0.05) compared to those on zidovudine, lamivudine and nevirapine [14]. Unfortunately, these studies were not designed to address a specific ART effect on helminth infections. Anthelminthic drugs of the benzimidazole class appear to act on the mitochondrion, supposedly on its tubulin structure [15]. Interestingly, mitochondrial toxicity seems to be a common adverse effect of many anti-retroviral drugs [16], with stavudine exhibiting the highest degrees of mitochondrial toxicity [17]. This toxic effect is mediated through multiple mitochondrial pathways including inhibition of gamma DNA polymerase [18].

Whether CTX is effective against helminths is less clear. It consists of sulfamethoxazole and trimethoprim which interfere with folate biosynthesis and metabolism. CTX is active against various bacteria, *Pneumocystis jirovecii*, several parasites, such as *Toxoplasma gondii* and *Plasmodium* spp. [19,20], and the intestinal parasites *Isospora belli and Cyclospora cayetanensis* [20].

The aims of this study were to systematically assess the prevalence of helminth infection among HIV-patients in Lambaréné, Gabon, and to investigate whether ART or CTX-P use is associated with a reduced risk of helminth infections. We hypothesized (i) that ART may be associated with a reduced risk of helminth infection, possibly due to mitochondrial toxicity of the ART on the worms, and that (ii) CTX-P may reduce the risk of helminth infection through intervention with the folic acid metabolism of the worms.

## Materials and Methods

### Study site

Between October 2012 and June 2014, a cross-sectional study was conducted in Lambaréné, a town of 25,000 inhabitants situated within the Central African rainforest area of the Moyen Ogooué province, Gabon. Patients were recruited at the Centre de Traitement Ambulatoire (CTA), which is the main clinic for HIV-care in Lambaréné. At this clinic, patients were followed up every 3 months, with an additional follow-up visit after 2 weeks if a patient was started on ART. ART was initiated if patients had CD4 counts <350 cells/μL or were symptomatic (World Health Organization (WHO) stage 3 or 4 [21]). Patients received CTX-P if they had CD4 counts <200 cells/μL, in accordance with the Gabonese national guidelines valid at the time of the study. Cotrimoxazole was stopped after two subsequent measurements of CD4 count above 200 cells/uL. Diagnostics for intestinal parasites were not routinely done. However, patients did receive a single dose of albendazole on a 3 monthly basis. Parasitologic diagnostic procedures were performed at the Centre de Recherches Médicales de Lambaréné (CERMEL). The study design capitalized on the limited data available; namely a prevalence of helminth infections of 40–50% in pregnant women and 70% in school-going children [22,23]. Filariases and schistosomiasis are endemic in the region [23,24]. *S*. *haematobium* is endemic in Gabon, whereas *S*. *mansoni* is not. All hookworm infections are *N*. *americanus; A*. *duodenale* is not endemic in the study area. HIV seroprevalence is estimated at 3.9% in Gabon [25].

### Ethics statement

Ethical clearance was obtained from the Institutional Research Board of CERMEL. All adult subjects provided written informed consent.

### Study population

Any consenting HIV-infected adult (age >18 years) attending the HIV clinic was invited to participate. Patients were divided into two groups: ART naïve, or taking ART for at least 3 months. Patients who started ART within 3 months before recruitment were excluded, to avoid potential effects from previous ART and CTX-P on helminths. Inclusion rates and CD4 counts of patients not enrolled were documented to avoid selection bias. Sample size was calculated based on a power of 80%, and an estimated 25% difference in prevalence between respective groups. We assumed that 65% of patients in the non-exposed group would be ‘helminth-infected’, based on available data [22–24]. The calculated sample size was 125 patients. However, an interim analysis revealed lower helminth infection prevalences (ART naïve 32%, on ART 19%). The sample size was amended accordingly to 352 patients, with some additional patients to compensate for patients submitting incomplete samples.

### Collection of data

Basic demographic data (age, sex, salary, profession, educational level, residence, pregnancy) were obtained and clinical data were collected using patient files (ART and CTX-P history, WHO stage [21], history of opportunistic infections, anthelminthic treatment) and laboratory registers from the HIV clinic (CD4 counts and hemoglobin). Three stool and urine samples were collected on consecutive days. Where possible, patients submitted stool samples on the day of collection. However, as many patients were living far away from the clinic, we used a maximum time period for acceptance of samples of 24 hours. Stool samples were examined for presence of eggs in smears prepared by the Kato Katz method [26]. Stool samples were analysed for the presence of larvae of *S*. *stercoralis* and *N*. *americanus* using the modified agar-plate culture technique [27]. Stool was incubated at 25°C on an agar plate. The culture supernatant was checked for larvae with microscopy after 3 and 7 days.

Urine was filtrated and analysed for presence of eggs by microscopy. In addition, 2 mL of blood was collected by venipuncture into EDTA around noon (11 am—1 pm). EDTA blood was analysed by direct microscopy. In addition, red blood cells were lysed using Saponin solution, after which cells were centrifuged. The pellet was analysed for presence of microfilariae by microscopy. If microfilariae were present, samples were stained with methylene blue to identify *Loa loa* or *M*. *perstans* [28]. All positive samples were confirmed by a senior laboratory technician.

### Outcomes and variables

Outcome was helminth infection, defined as at least one positive stool, urine or blood sample for intestinal helminths (*T*. *trichiura*, *A*. *lumbricoides*, *N*. *americanus*, *S*. *stercoralis)*, *S*. *haematobium* or microfilaria (*Loa loa* or *M*. *perstans*). Factors assessed for their association with helminth infection prevalence were ART and CTX-P use. As pre-defined risk factors were considered age, sex, educational level and rural versus urban residence. The following factors were considered as potential confounders: income, pregnancy, WHO stage, body mass index (BMI), CD4 count, hemoglobin and use of anthelminthic treatment <12 weeks prior to participation.

The outcome for the primary analysis was the diagnosis of any helminth infection. Patients were included if they provided at least 2 stool and urine samples and one blood sample.

Secondly, sub-analyses were done for different groups of helminth infections; intestinal helminths (*T*.*trichiura*, *A*. *lumbricoides*, *N*. *americanus* or *S*. *stercoralis*); *S*. *haematobium*; and the filariae *Loa loa* and *M*. *perstans*. Patients were included for sub-analyses if they provided at least 2 stool samples (intestinal helminths), 2 urine samples (*S*. *haematobium*) or 1 blood sample (filariases).

### Statistical analysis

The distribution of pre-defined risk factors, potential confounders and exposure to ART or CTX-P of patients who were diagnosed with any helminth infection were compared to those who were not infected. We used the χ² test for categorical data, the Student’s t-test for normally distributed continuous data and the Mann-Whitney-U test for non-normally distributed data.

Data were assessed for completeness. If for a certain factor, >10% of data were missing, patient characteristics for the group with missing data were compared to those with complete data.

Multivariable logistic regression analysis was used to assess the odds of helminth infection associated with the main exposures (ART and CTX-P). Odds ratios were adjusted for pre-defined risk factors. Potential confounders were assessed for their interaction with the main exposures (ART and CTX-P). Factors causing an odds ratio change of >10% were considered as confounders and included in the final model, avoiding multi-collinearity. CD4 count was excluded from the primary analysis because it is a key parameter in decision-making on ART initiation. CTX-P use is inevitably linked with ART use (patients on CTX-P will as well qualify for ART, unless ART naïve and treated for another opportunistic infection before starting ART). Therefore, we performed a sub-group analysis including only patients on ART to assess the odds of helminth infections associated with CTX-P exposure. In this same sub-group, the median time on ART was compared between patients diagnosed with helminth infections and those without using the Mann Whitney U test. We also included the time on ART in the multivariate analysis. Analyses were done using SPSS Statistics Version 21 (IBM, Chicago, IL, USA).

## Results

**Fig 1:**
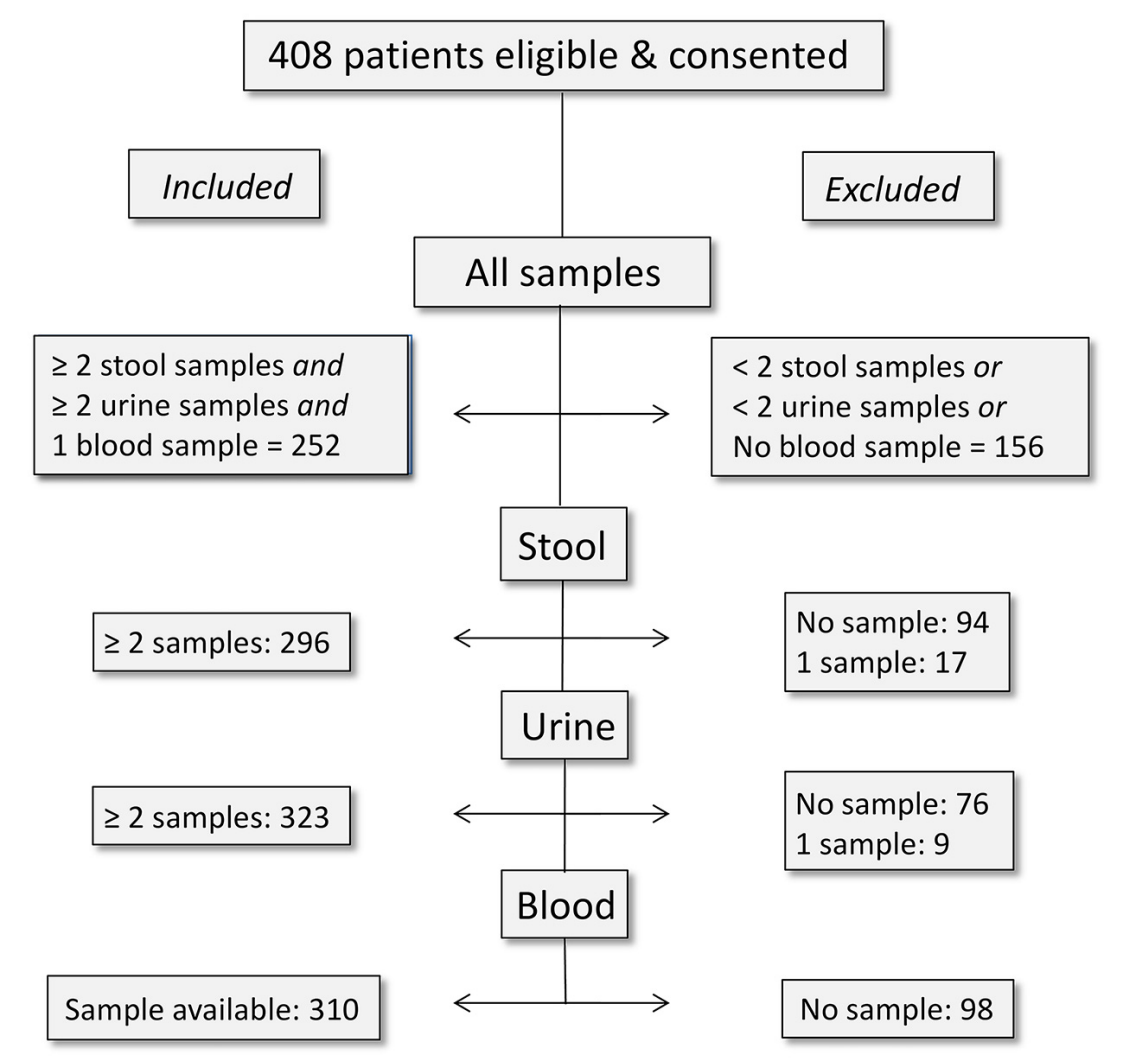
Study flow. Study flow displaying the inclusion of study participants. Patients were included in the main analysis if they provided at least 2 stool and urine samples and one blood sample. Patients were included for subanalyses if they provided at least 2 stool samples (intestinal helminths), 2 urine samples (*S*. *haematobium*) or 1 blood sample (filariases).

A total of 803 patients were screened and 408 patients were recruited (Fig 1). Two-hundred fifty-two patients submitted at least 2 stool and urine samples and one blood sample, and were included in the main analysis.

Baseline characteristics of the study population are given in Table 1. The mean age was 41 years (standard deviation (SD) 12 yrs); the majority was female. The majority of patients lived in a semi-urban setting, and had a highest educational level of secondary school. The median CD4 count at the time of study participation was 356 cells/μL (interquartile range [IQR] 186–526). Around 60% of patients had been on ART for at least 3 months. Almost half the study population was receiving CTX-P.

**Table 1:** Patient characteristics.

	Data^a^ (n)	Total cohort	Not infected	Any infection	P-value^b^
Age; years (mean, SD^c^)	250	41.9 (12.0)	42.6 (12.4)	40.3 (10.9)	0.18
Sex (female)(n, %)	252	169 (67.1)	127 (72.6)	42 (54.5)	0.005
*Residence* (n,%)	249				0.004
- Rural		76 (30.5)	43 (24.7)	44.0)	
- Semi-urban		148 (59.4)	111 (63.8)	49.3)	
- Urban		25 (10.0)	20 (11.5)	5 (6.7)	
*Educational level* (n, %)	243				0.07
- Lower than primary		23 (9.5)	15 (8.9)	(10.8)	
- Primary		66 (27.2)	40 (23.7)	35.1)	
- Secondary		8.3)	108 (63.9)	52.7)	
- Tertiary/higher		7 (2.8)	6 (3.6)	1 (1.4)	
CD4 count; cells/μL (median, IQR^d^)	247	366 (177–555)	345(159–531)	382(185–579)	0.85
*CD4 count stratified* (n,%)					0.99
- 0–100 cells/μL		36 (14.6)	21 (12.4)	19.5)	
- 101–200 cells/μL		33 (13.4)	27 (15.9)	(7.8)	
- 201–350 cells/μL		51 (20.6)	38 (22.4)	16.9)	
- >351 cells/μL		127 (51.4)	84 (49.4)	43 (55.8)	
Hemoglobin; g/dL (median, IQR^d^)	160	10.0 (10.0–12.0)	11.0 (10.0–12.0)	11.7 (10.7–12.7)	0.07
On ART^e^ >12 weeks (n, %)	252	149 (59.1)	111 (63.4)	38 (49.4)	0.04
On CTX-P^f^ (n, %)	222	103 (46.4)	76 (47.8)	27 (42.9)	0.51
Antihelminth treatment <12 weeks (n, %)	151	53 (35.1)	38 (36.5)	15 (31.9)	0.58

Characteristics of those patients who were diagnosed with one or more helminth infections versus those who had negative test results.

^a ^The first column shows for how many patients data were complete for each respective variable.

^b^ P-values were calculated using the χ² test was used for categorical variables (ordinal χ² test if more than 2 categories), the Students' T test for linear normally distributed variables, and Mann Whitney U for non-parametric variables.

^c^ Standard deviation (SD),

^d^ Interquartile range (IQR),

^e ^Anti-retroviral therapy (ART),

^f^ Cotrimoxazole preventive therapy (CTX-P).

For most determinants, completeness of data was >90%. However, there was >10% missing data for hemoglobin (92/252, 36%), the self-reported use of anthelminthic treatment (101/252, 40.1%), and use of CTX-P (30/252, 11.9%). Patient characteristics for missing data are given in S1 Table. ART use was reported less often for patients with missing data for all 3 variables. Patients with missing data on the use of CTX-P were more likely to have any infection and to have an infection with intestinal helminths.

**Fig 2:**
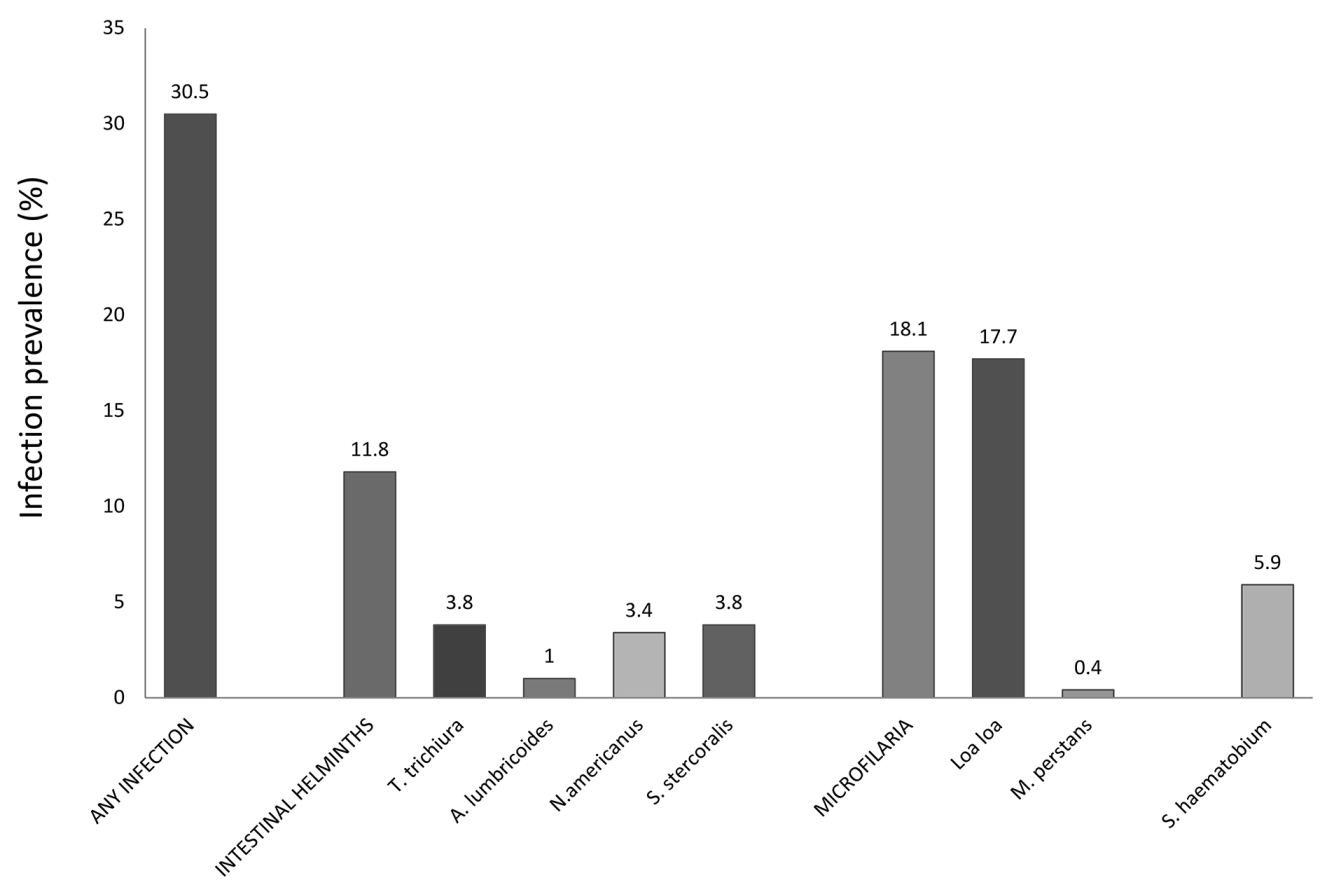
Prevalence of helminth infections. The prevalence of helminth infections is displayed for all infections together, as well as the subgroups intestinal helminth infections and filariases, and for each individual infection.

Fig 2 displays the prevalence of helminth infections. The overall prevalence was 77/252 (30.5%). Sub-analyses showed that filariases were most prevalent (56/310, 18.1%); 19/323 (5.9%) individuals were infected with *S*. *haematobium;* 35/296 (11.8%) subjects carried one or more intestinal helminths.

Patients carrying one or more helminths were more frequently male, lived in rural areas and were less educated (Table 1). There were no differences in income, WHO stage, pregnancy or BMI (S2 Table).

Patient characteristics for patients infected with intestinal helminths or *Loa loa* versus those not infected are given in S3 Table. Patients diagnosed with intestinal helminths were more often living in rural areas as compared to those with no diagnosis of intestinal helminths. *Loa loa*, the most prevalent infection in this cohort, was found more frequently in male patients, and ART and CTX-P use were reported more often in the non-infected patient group.

In multivariable logistic regression, there was no evidence of an association between ART use and the risk of having any infection, infection with intestinal helminths, or *Loa loa* (Table 2). In contrast, CTX-P was associated with a reduced risk of *Loa loa* microfilaremia, in the whole population as well as in the subgroup analysis of patients on ART. Female sex was associated with a 3-fold decreased risk of any infection and loiasis. Lower education level was associated with a 2.5-fold increased risk of having any infection, and rural residence was associated with an almost 4-fold increased risk of infection with one or more intestinal helminths. There was no evidence of an association between time on ART and the risk of having any infection, infection with intestinal helminths, or *Loa loa* (Table 2). Also, there was no difference in median time on ART of patients diagnosed with one or more helminth infections compared to those not infected (S4 Table).

**Table 2:** Effect of exposure to ART or CTX-P on helminth infection prevalence.

	Any infection			Intestinal helminths			*Loa loa*		
***Whole study population***	aOR	95% CI	P-value	aOR	95% CI	P-value	aOR	95% CI	P-value
ART^a^	0.88	0.45–1.73	0.71	0.67	0.27–1.61	0.37	0.56	0.29–1.11	0.10
Sex^1^	0.26	0.12–0.55	<0.001	0.60	0.22–1.61	0.31	0.33	0.16–0.69	0.003
Age^2^	0.64	0.27–0.87	0.005	0.76	0.51–1.12	0.16	0.80	0.60–1.08	0.15
Educational level^3^	2.39	1.18–4.85	0.02	2.05	0.80–5.28	0.14	1.41	0.67–2.94	0.36
Residence^4^	2.04	1.03–4.03	0.04	3.65	1.48–8.98	0.005	1.63	0.81–3.31	0.17
CTX-P^b^	0.78	0.41–1.73	0.71	0.47	0.29–1.19	0.11	0.47	0.23–0.97	0.04
***Subgroup on ART*** ^***a***^									
CTX-P^b^	1.04	0.44–2.52	0.91	0.75	0.22–2.54	0.64	0.34	0.12–0.92	0.03
Sex^1^	0.29	0.10–0.80	0.02	1.05	0.25–4.30	0.94	0.18	0.06–0.56	0.003
Age^2^	0.63	0.40–0.98	0.04	0.96	0.50–1.82	0.89	0.70	0.43–1.13	0.14
Educational level^3^	3.18	1.24–8.13	0.02	2.48	0.71–8.63	0.15	2.42	0.85–6.94	0.10
Residence^4^	1.41	0.56–3.52	0.46	5.68	1.54–20.9	0.009	0.64	0.22–1.85	0.41
CD4 count ^5^	1.01	0.92–1.10	0.84	0.89	0.77–1.04	0.15	1.08	0.98–1.19	0.11
Time on ART^6^ ^a^	1.01	1.00–1.03	0.15	1.00	0.97–1.02	0.88	0.99	0.97–1.01	0.31

Multivariable logistic regression analysis was used to assess the odds of helminth infections associated with the main exposures (ART and CTX-P). Odds ratios were adjusted for pre-defined risk factors. Potential confounders were assessed for their interaction with the main exposures (ART and CTX-P). Factors causing an odds ratio change of >10% were considered as confounders and included in the final model, avoiding multi-collinearity.

^1^Female versus male sex,

^2 ^age per 10 years increase,

^3^ primary school or lower versus secondary school or higher,

^4^rural residence versus (semi-)urban,

^5^ CD4 count per 50 cells/μL increase,

^6 ^time on ART in months,

^a ^Anti-retroviral therapy (ART),

^b^ Cotrimoxazole preventive therapy (CTX-P)

## Discussion

This study assessed the prevalence of helminth infections in HIV-infected adults in Lambaréné, Gabon, and the association of ART and CTX-P with the prevalence of helminth infections. ART use did not alter the risk of harbouring any of the studied infections; intestinal helminths (*T*. *trichiura*, *A*. *lumbricoides*, *N*. *americanus*, *S*. *stercoralis*), *S*. *haematobium*, or microfilaria (*Loa loa*, *M*. *perstans*). In contrast, CPT use reduced the risk of *Loa loa* microfilaremia. The overall infection prevalence was 30.5%, which compares favorably to earlier studies in the same region [22–24], yet is well in line with similar studies from other settings [9–13]. The recruitment of individuals who may have a different risk for helminth infections, like pregnant women or school children, hampered direct study comparisons. Furthermore, many studies investigated only intestinal parasites but included protozoa [9–14]. In this study, systemic helminth infections were also investigated, with filarial infections being most prevalent (18.1%) followed by *S*. *haematobium* (5.9%). Intestinal helminth infections were found in only 11.8%, in line with infection rates observed in other studies [13,14]. However, higher rates for *A*. *lumbricoides* and *S*. *stercoralis* have been reported, especially in the pre-ART era [9].

This study found microfilaremia more frequently in males. This finding is not new [24,29]. One possible explanation is behavioral factors, such as male patients working in the forest and therefore being more exposed during day time [24,29]. Animal models have shown potential effects of gonadal hormones [30]. Rural residence is a known risk factor for helminth infection [31,32] and was associated with infections in this study. In general, factors associated with poverty are associated with an increased risk for helminth infection [31,32].

CD4 counts were not correlated with the risk of harboring helminth infections. No significant difference in CD4 count distribution was observed between helminth-infected and uninfected patients (Table 1). Only one hyper-infection syndrome with *S*. *stercoralis* was encountered in a HTLV-1 co-infected patient [33]. Data on the interplay of immune depression due to HIV and helminth infections are conflicting. A recent study reported higher co-infection risk with higher CD4 counts [31]. In agreement with this, a negative association of CD4 counts and risk of helminth infection was reported in HIV-infected patients in Uganda [34]. However, conflicting data have been reported from other settings [32]. None of these studies documented CTX-P as a potential confounder or causal factor for the altered prevalence of helminth infection in patients with lower CD4 counts.

Although several studies addressed the effect of deworming on HIV viral load and CD4 counts as reviewed elsewhere [35,36], little is known about the opposite effect of HIV-related treatments such as ART and CTX-P on helminths. In this study, ART use did not alter the risk of any helminth infections. However, although ART was not associated with a risk reduction of helminth infections when adjusted to pre-defined risk factors and confounders, the rather low number of infections may have masked a more discrete effect. In contrast, CTX-P reduced the risk of *Loa loa* microfilaremia in the whole population as well as in the subgroup of patients on ART (Table 2).

Antifilarial effects of dihydrofolate reductase (DHFR) inhibitors seem to exist for lymphatic filariae [37], but no data are available for *Loa loa* and *M*. *perstans*. The present study suggests CTX-P may have antifilarial effects on *Loa loa* microfilaria, most likely by acting as a DHFR inhibitor thereby inducing apoptosis of the microfilaria [37,38]. The low number of cases of *M*. *perstans* and *S*. *haematobium* infection did not allow assessment of this association, and further exploration seems warranted. CTX-P might be a safer alternative compared to the currently available agents, DEC and ivermectin, either as curative agent, or used for reducing worm burden prior to administration of one of the classical drugs to reduce the risk of adverse reactions.

Interestingly, a potentially reduced risk of infection with intestinal helminths in patients receiving CTX-P was not observed. One explanation could be the lower trimethoprim affinity to intestinal helminths’ DHFR, while lower bioavailability and drug concentrations at the site of intestinal worm infection may be alternative explanations. In fact, CTX is very well absorbed and mainly excreted in urine [39] and thus levels in the gut may be too low to be effective against helminths. However, CTX is used successfully to treat several intestinal infections, caused by bacteria and protozoa. They might be more sensitive even to lower concentrations, considering that their DHFR has a much higher affinity to trimethoprim than intestinal helminths.

This study has several strengths. To increase specificity of the parasitologic diagnosis, at least 2 negative stool or urine samples were necessary to qualify as a negative test result. The Saponin method for detection of microfilariae has been found superior to a thick blood smear [40]. However, the absence of microfilaraemia does not exclude infection with *Loa loa* or *M*. *perstans*. Previously reported risk factors were found also in this study, thereby assuring the external validity of the data reported.

However, there are also limitations. Although recommended by the WHO, the sensitivity of the Kato-Katz method is not optimal [41]. The modified agar-plate culture method is relatively sensitive for detection of *S*. *stercoralis* larvae, but less so for hookworm larvae [42]. It should be noted that not all studies use the same methodology for the detection of parasites in stools, which may make direct comparisons difficult, especially at low concentrations of eggs. By using more sensitive methods, possibly more cases could have been identified, although it appears doubtful that this would have changed the overall results. Secondly, not all patients submitted sufficient samples to be included in the final analysis. Therefore, the statistical power to detect certain associations was lower than expected even though the calculated sample size was reached. The use of anthelminthic treatment was not directly observed and self-reported data available on its use during the 12 weeks prior to study participation were limited. There was no clearly reduced infection prevalence in patients who reported prior anthelminthic treatment. This could be explained by the fact that the anthelminthic treatment which was administered at the time of the study was single-dose albendazole, of which the efficacy has been shown to be limited in this setting [43]. Recall bias may have played a role in data availability on self-reported anthelminthic treatment. Prevalences of helminth infections did not differ among patients with incomplete data on the use of antihelminth treatment versus those with complete data, although they were more likely to have certain risk factors for infection (S1 Table).

Drugs with less mitochondrial toxicity might have been used in the treatment scheme, thus masking any anti-helminthic effect of ART based on mitochondrial toxicity. The design of this study did not permit assessment of associations of helminth infection with different antiretroviral drugs. In the absence of randomisation between treatment regimens, analyses of treatment effects in observational data are possible using causal methods such as inverse probability weighting, for which our sample was too small. Also, we did not collect data on compliance to ART and cotrimoxazole. Lack of compliance in certain patient groups may have masked anti-helminthic effects of both ART and cotrimoxazole. There were no reliable data available on the duration of CTX-P. Therefore a sensitivity analysis of the effect of the duration of CTX-P on the risk of helminth infections was not possible.

In conclusion, the main findings of this study are a high prevalence of *Loa loa* microfilaremia in HIV-patients in Gabon, and a decreased risk of *Loa loa* infection in patients using CTX-P, suggesting an anthelminthic effect of antifolate drugs. No association of ART use and helminth infections was established. Additional studies are needed to further assess the effects of CTX on blood-dwelling microfilariae and other helminths, as this might be a safe, cheap and effective alternative to (few) existing treatment options.

## Supporting Information

S1 Checklist STROBE checklist completed for this manuscript. (DOCX)

S1 Table Patient characteristics of patients with missing data compared to those with complete data. (DOCX)

S2 Table Patient characteristics, continued. Patient characteristics of patients who were diagnosed with one or more helminth infections versus those who had negative test results.
(DOCX)

S3 Table Patient characteristics comparing participants infected versus non-infected with intestinal helminths and *Loa loa*. (DOCX)

S4 Table Time on ART for participants infected versus non-infected with one or more helminth infections, intestinal helminths or *Loa loa*. (DOCX)

S1 Dataset SPSS Statistics database containing all data used for analysis. (SAV)
