# Supplementary material for: Impact of Anti-Retroviral Treatment and Cotrimoxazole Prophylaxis on Helminth Infections in HIV-Infected Patients in Lambaréné, Gabon
Source: PLoS Negl Trop Dis. 2015 May 20;9(5):e0003769. doi: 10.1371/journal.pntd.0003769 (PMC4439024; doi:10.1371/journal.pntd.0003769)
Supplement: S1 Checklist — (DOCX) [file pntd.0003769.s001.docx]

**STROBE Statement—checklist of items that should be included in reports of observational studies**

**Paper: Janssen et al. Impact of Anti-Retroviral Treatment and Cotrimoxazole Prophylaxis on Helminth Infections in HIV-Infected Patients in Lambaréné, Gabon**

|  | | | Item No. | Recommendation | Page  No. | | | Relevant text from manuscript |
| --- | --- | --- | --- | --- | --- | --- | --- | --- |
| **Title and abstract** | | | 1 | (*a*) Indicate the study’s design with a commonly used term in the title or the abstract | 3 | | | Line 9 |
|  |  |  |  | (*b*) Provide in the abstract an informative and balanced summary of what was done and what was found | 3 | | | Lines 9-19 |
| Introduction | | | | | | | |  |
| Background/rationale | | | 2 | Explain the scientific background and rationale for the investigation being reported | 4-6 | | | Lines 46-76 |
| Objectives | | | 3 | State specific objectives, including any prespecified hypotheses | 6 | | | Lines 77-82 |
| Methods | | | | | | | |  |
| Study design | | | 4 | Present key elements of study design early in the paper | 3, 6-8 | | | Lines 9-13  Line 85  Lines 100-137 |
| Setting | | | 5 | Describe the setting, locations, and relevant dates, including periods of recruitment, exposure, follow-up, and data collection | 6 | | | Lines 84-96 |
| Participants | | | 6 | (*a*) *Cohort study*—Give the eligibility criteria, and the sources and methods of selection of participants. Describe methods of follow-up  *Case-control study*—Give the eligibility criteria, and the sources and methods of case ascertainment and control selection. Give the rationale for the choice of cases and controls  *Cross-sectional study*—Give the eligibility criteria, and the sources and methods of selection of participants | 7 | | | Lines 100-105 |
|  |  |  |  | (*b*) *Cohort study*—For matched studies, give matching criteria and number of exposed and unexposed  *Case-control study*—For matched studies, give matching criteria and the number of controls per case |  | | |  |
| Variables | | | 7 | Clearly define all outcomes, exposures, predictors, potential confounders, and effect modifiers. Give diagnostic criteria, if applicable | 7-8 | | | Lines 124-137 |
| Data sources/ measurement | | | 8* | For each variable of interest, give sources of data and details of methods of assessment (measurement). Describe comparability of assessment methods if there is more than one group | 7 | | | Lines 110-123 |
| Bias | | | 9 | Describe any efforts to address potential sources of bias | 7,8 | | | Lines 104-105  Lines 122-123  Lines 143-144 |
| Study size | | | 10 | Explain how the study size was arrived at | 7 | | | Lines 105-109 |
| Quantitative variables | | 11 | | Explain how quantitative variables were handled in the analyses. If applicable, describe which groupings were chosen and why | 8,10,13 | | Lines 139-140  Lines 200-201 | |
| Statistical methods | | 12 | | (*a*) Describe all statistical methods, including those used to control for confounding | 8,9 | | Lines 138-154 | |
|  |  |  |  | (*b*) Describe any methods used to examine subgroups and interactions | 8,9 | | Lines 150-154 | |
|  |  |  |  | (*c*) Explain how missing data were addressed | 8 | | Lines 143-144  Supplementary Table 1 | |
|  |  |  |  | (*d*) *Cohort study*—If applicable, explain how loss to follow-up was addressed  *Case-control study*—If applicable, explain how matching of cases and controls was addressed  *Cross-sectional study*—If applicable, describe analytical methods taking account of sampling strategy | n/a | | n/a | |
|  |  |  |  | (*e*) Describe any sensitivity analyses | n/a | | n/a | |
| Results | | | | | | | | |
| Participants | | 13* | | (a) Report numbers of individuals at each stage of study—eg numbers potentially eligible, examined for eligibility, confirmed eligible, included in the study, completing follow-up, and analysed | 9 | | Lines 156-158  Figure 1 Study Flow | |
|  |  |  |  | (b) Give reasons for non-participation at each stage |  | | Figure 1 Study Flow | |
|  |  |  |  | (c) Consider use of a flow diagram |  | | Figure 1 Study Flow | |
| Descriptive data | | 14* | | (a) Give characteristics of study participants (eg demographic, clinical, social) and information on exposures and potential confounders | 9-11 | | Lines 159-168 | |
|  |  |  |  | (b) Indicate number of participants with missing data for each variable of interest | 10,11 | | Lines 169-172  Table 1  Supplementary Table 1 | |
|  |  |  |  | (c) *Cohort study*—Summarise follow-up time (eg, average and total amount) | n/a | | n/a | |
| Outcome data | | 15* | | *Cohort study*—Report numbers of outcome events or summary measures over time | n/a | | n/a | |
|  |  |  |  | *Case-control study—*Report numbers in each exposure category, or summary measures of exposure | n/a | | n/a | |
|  |  |  |  | *Cross-sectional study—*Report numbers of outcome events or summary measures | 11 | | Lines 173-176  Figure 2 | |
| Main results | | 16 | | (*a*) Give unadjusted estimates and, if applicable, confounder-adjusted estimates and their precision (eg, 95% confidence interval). Make clear which confounders were adjusted for and why they were included | 12-14 | | Lines 185-202 | |
|  |  |  |  | (*b*) Report category boundaries when continuous variables were categorized | 13 | | Lines 200-201 | |
|  |  |  |  | (*c*) If relevant, consider translating estimates of relative risk into absolute risk for a meaningful time period | n/a | | n/a | |
| Other analyses | 17 | | Report other analyses done—eg analyses of subgroups and interactions, and sensitivity analyses | | 12-14 | Lines 186-201 | | |
| Discussion | | | | | | | | |
| Key results | 18 | | Summarise key results with reference to study objectives | | 14 | Lines 205-211 | | |
| Limitations | 19 | | Discuss limitations of the study, taking into account sources of potential bias or imprecision. Discuss both direction and magnitude of any potential bias | | 16,17 | Lines 262-276 | | |
| Interpretation | 20 | | Give a cautious overall interpretation of results considering objectives, limitations, multiplicity of analyses, results from similar studies, and other relevant evidence | | 14-16 | Lines 205-255 | | |
| Generalisability | 21 | | Discuss the generalisability (external validity) of the study results | | 14,16 | Lines 214-216  Lines 259-261 | | |
| Other information | | |  | | | | | |
| Funding | 22 | | Give the source of funding and the role of the funders for the present study and, if applicable, for the original study on which the present article is based | | n/a | In online submission | | |

*Give information separately for cases and controls in case-control studies and, if applicable, for exposed and unexposed groups in cohort and cross-sectional studies.

**Note:** An Explanation and Elaboration article discusses each checklist item and gives methodological background and published examples of transparent reporting. The STROBE checklist is best used in conjunction with this article (freely available on the Web sites of PLoS Medicine at http://www.plosmedicine.org/, Annals of Internal Medicine at http://www.annals.org/, and Epidemiology at http://www.epidem.com/). Information on the STROBE Initiative is available at www.strobe-statement.org.
