# Supplementary material for: Impact of Anti-Retroviral Treatment and Cotrimoxazole Prophylaxis on Helminth Infections in HIV-Infected Patients in Lambaréné, Gabon
Source: PLoS Negl Trop Dis. 2015 May 20;9(5):e0003769. doi: 10.1371/journal.pntd.0003769 (PMC4439024; doi:10.1371/journal.pntd.0003769)
Supplement: S1 Table — (DOCX) [file pntd.0003769.s002.docx]

**S1 Table 1. Patient characteristics of patients with missing data compared to those with complete data**

|  | **Hemoglobin** | |  | **Anthelminthic treatment** | | | **Cotrimoxazole Preventive Treatment (CPT-X)** | | |
| --- | --- | --- | --- | --- | --- | --- | --- | --- | --- |
| **Variables** | **Complete (n=160)** | **Missing (n=92)** | **P-value^a^** | **Complete (n=151)** | **Missing (n=101)** | **P-value^a^** | **Complete (n=222)** | **Missing (n=30)** | **P-value^a^** |
| Age; years (mean, SD^b^) | 41.2 (11.4) | 43.0 (12.9) | 0.23 | 42.4 (11.4) | 41.1 (12.9) | 0.39 | 42.0 (12.1) | 41.2 (11.4) | 0.75 |
| Sex (female) | 110 (68.8) | 59 (64.1) | 0.45 | 96 (63.6) | 73 (72.3) | 0.15 | 147 (66.2) | 22 (73.3) | 0.44 |
| *Residence* (n,%) |  |  |  |  |  |  |  |  |  |
| Rural | 46 (29.1) | 30 (33.0) | 0.89 | 40 (26.7) | 36 (36.4) | 0.003 | 67 (30.5) | 9 (31.0) | 0.50 |
| Semi-urban | 99 (62.7) | 49 (53.8) |  | 87 (58.0) | 61 (61.6) |  | 129 (58.6) | 19 (65.5) |  |
| Urban | 13 (8.2) | 12 (13.2) |  | 23 (15.3) | 2 (2.0) |  | 24 (10.9) | 1 (3.4) |  |
| *Educational level* (n,%) |  |  |  |  |  |  |  |  |  |
| None | 14 (9.2) | 9 (10.0) | 0.69 | 12 (8.1) | 11 (11.7) | 0.05 | 21 (9.8) | 2 (7.1) | 0.90 |
| Primary | 41 (26.8) | 25 (27.8) |  | 36 (24.2) | 30 (31.9) |  | 57 (26.5) | 9 (32.1) |  |
| Secondary | 93 (60.8) | 54 (60.0) |  | 95 (63.8) | 52 (55.3) |  | 131 (60.9) | 16 (57.1) |  |
| Tertiary/higher | 5 (3.3) | 2 (2.2) |  | 6 (4.0) | 1 (1.1) |  | 6 (2.8) | 1 (3.6) |  |
| CD4 count; cells/µL (median, IQR^c^) | 357 (188-526) | 381 (175-587) | 0.33 | 381 (189-574) | 341 (168-509) | 0.50 | 345 (170-520) | 451 (181-722) | 0.10 |
| Hemoglobin; g/dL  (median, IQR^c^) | - | - | - | 11.3 (10.3-12.3) | 10.6 (9.6-11.6) | 0.003 | 11.1 (10.1-12.1) | 10.8 (9.8-11.8) | 0.15 |
| On ART^d^ >12 weeks (n,%) | 105 (65.6) | 44 (47.8) | 0.008 | 109 (72.2) | 40 (39.6) | <0.001 | 141 (63.5) | 8 (26.7) | <0.001 |
| On CTX-P^e^ (n,%) | 72 (50.0) | 31 (39.7) | 0.14 | 61 (44.9) | 42 (48.8) | 0.56 | - | - | - |
| Anthelminthic treatment <12 weeks (n,%) | 37 (36.3) | 16 (32.7) | 0.66 | - | - | - | 43 (31.6) | 10 (66.7) | 0.007 |
| Any infection (n,%) | 48 (30.0) | 29 (31.5) | 0.80 | 47 (31.1) | 30 (29.7) | 0.81 | 63 (28.4) | 14 (46.7) | 0.04 |
| Intestinal helminths (n,%) | 18 (11.3) | 13 (14.1) | 0.50 | 16 (10.6) | 15 (14.9) | 0.31 | 23 (10.4) | 8 (26.7) | 0.01 |
| *Loa loa* (n,%) | 23 (14.4) | 20 (21.7) | 0.14 | 27 (17.9) | 16 (15.8) | 0.67 | 37 (16.7) | 6 (20.0) | 0.65 |

Data were assessed for completeness. If for a certain factor, >10% of data were missing, patient characteristics for the group with missing data were compared to those with complete data.

^a^P-values were calculated using the χ² test was used for categorical variables (ordinal χ² test if more than 2 categories), the Students' T test for linear normally distributed variables, and Mann Whitney U for non-parametric variables.

^b^ Standard deviation (SD), ^c^ Interquartile range (IQR), ^d^ Anti-retroviral therapy (ART), ^e^ Cotrimoxazole preventive therapy (CTX-P)
