# Supplementary material for: Impact of Anti-Retroviral Treatment and Cotrimoxazole Prophylaxis on Helminth Infections in HIV-Infected Patients in Lambaréné, Gabon
Source: PLoS Negl Trop Dis. 2015 May 20;9(5):e0003769. doi: 10.1371/journal.pntd.0003769 (PMC4439024; doi:10.1371/journal.pntd.0003769)
Supplement: S2 Table — Patient characteristics of patients who were diagnosed with one or more helminth infections versus those who had negative test results. (DOCX) [file pntd.0003769.s003.docx]

| **S2 Table 2. Patient characteristics - continued** | | | | | |
| --- | --- | --- | --- | --- | --- |
|  | **Data^a^ (n)** | **Total cohort** | **Not infected** | **Any infection** | **P-value^b^** |
| BMI^c^ (median, IQR^d^) | 225 | 21.6 (19.0-24.2) | 21.7 (18.5-24.8) | 21.5 (19.6-23.5) | 0.78 |
| WHO^e^ stage (n,%) | 199 |  |  |  | 0.11 |
| 1 |  | 122 (61.3) | 93 (66.9) | 29 (48.3) |  |
| 2 |  | 29 (14.6) | 15 (10.8) | 14 (23.3) |  |
| 3 |  | 37 (18.6) | 23 (16.5) | 14 (23.3) |  |
| 4 |  | 11 (5.5) | 8 (5.8) | 3 (5.0) |  |
| *Income in CFA^f^ (n,%)* | 246 |  |  |  | 0.77 |
| - 0-20 000 |  | 136 (55.3) | 95 (53.6) | 41 (54.7) |  |
| - 20 000 – 40 000 |  | 58 (23.6) | 39 (22.8) | 19 (25.3) |  |
| - 40 000 – 60 000 |  | 37 (15.0) | 25 (14.6) | 12 (16.0) |  |
| - > 60 000 |  | 15 (6.1) | 12 (7.0) | 3 (4.0) |  |
| Pregnant at enrolment (n,%) | 252 | 9 (3.6) | 6 (3.4) | 3 (3.9) | 0.55 |
|  |  |  |  |  |  |

Patient characteristics of patients who were diagnosed with one or more helminth infections versus those who had negative test results.

^a^ The first column shows for how many patients data were complete for each respective variable.

^b^ P-values were calculated using the χ² test was used for categorical variables (ordinal χ² test if more than 2 categories), the Students' T test for linear normally distributed variables, and Mann Whitney U for non-parametric variables.

^c^ Body Mass Index (BMI), ^d^ Interquartile range (IQR), ^e^ World Health Organization (WHO), ^f^ Central African Franc (CFA); 1 USD = 550 CFA.
