# Supplementary material for: Impact of Anti-Retroviral Treatment and Cotrimoxazole Prophylaxis on Helminth Infections in HIV-Infected Patients in Lambaréné, Gabon
Source: PLoS Negl Trop Dis. 2015 May 20;9(5):e0003769. doi: 10.1371/journal.pntd.0003769 (PMC4439024; doi:10.1371/journal.pntd.0003769)
Supplement: S3 Table — (DOCX) [file pntd.0003769.s004.docx]

**S3 Table 3. Patient characteristics comparing participants infected versus non-infected with intestinal helminths and *Loa loa***

|  | **Intestinal helminths** | | |  | ***Loa loa*** |  |  |  |
| --- | --- | --- | --- | --- | --- | --- | --- | --- |
|  | **Data (n)^a^** | **Not infected** | **Infected** | **P-value^b^** | **Data (n)^a^** | **Not infected** | **Infected** | **P-value^b^** |
| Age; years (mean, SD^c^) |  | 41.7 (11.8) | 41.2 (12.4) | 0.80 |  | 41.8 (12.3) | 42.3 (11.1) | 0.8 |
| Sex (female)(n, %) | 291 | 178 (69.5) | 20 (57.1) | 0.14 | 310 | 186 (72.9) | 27 (49.1) | 0.001 |
| *Residence* (n,%) | 288 |  |  |  | 307 |  |  |  |
| Rural |  | 66 (26.0) | 19 (55.9) | 0.009 |  | 70 (27.7) | 20 (37.0) | 0.06 |
| Semi-urban |  | 159 (62.6) | 11 (32.4) |  |  | 154 (60.9) | 32 (59.3) |  |
| Urban |  | 29 (11.4) | 3 (11.8) |  |  | 29 (11.5) | 2 (3.7) |  |
| *Educational level* (n, %) | 282 |  |  |  | 301 |  |  |  |
| Lower than primary |  | 22 (8.9) | 4 (11.8) | 0.12 |  | 23 (9.3) | 8 (14.8) | 0.45 |
| Primary |  | 62 (25.0) | 13 (38.2) |  |  | 70 (28.3) | 13 (24.1) |  |
| Secondary |  | 154 (62.1) | 16 (47.1) |  |  | 146 (59.1) | 32 (59.3) |  |
| Tertiary/higher |  | 10 (4.0) | 1 (2.9) |  |  | 8 (3.2) | 1 (1.9) |  |
| CD4 count; cells/µL (median, IQR^d^) | 285 | 345 (164-526) | 397 (178-616) | 0.48 | 302 | 351 (179-523) | 441 (224-658) | 0.31 |
| Hemoglobin; g/dL (median, IQR^d^) | 178 | 10.9 (9.9-11.9) | 11.7 (10.7-12.7) | 0.04 | 184 | 11.0 (10.0-12.0) | 11.9 (10.9-12.9) | 0.02 |
| On ART^e^ >12 weeks (n, %) | 291 | 164 (64.1) | 17 (48.6) | 0.08 | 310 | 166 (65.1) | 25 (45.5) | 0.02 |
| On CTX-P^f^ (n, %) | 257 | 111 (51.9) | 8 (30.8) | 0.09 | 275 | 109 (48.2) | 14 (28.6) | 0.01 |
| Antihelminth treatment <12 weeks (n, %) | 176 | 53 (33.3) | 5 (29.4) | 0.74 | 182 | 54 (36.5) | 8 (23.5) | 0.15 |
|  |  |  |  |  |  |  |  |  |

Patient characteristics of patients who were diagnosed with one or more intestinal helminths or *Loa loa* versus those who had negative test results.

^a^ The first column shows for how many patients data were complete for each respective variable.

^b^ P-values were calculated using the χ² test was used for categorical variables (ordinal χ² test if more than 2 categories), the Students' T test for linear normally distributed variables, and Mann Whitney U for non-parametric variables.

^c^ Standard deviation (SD), ^d^ Interquartile range (IQR), ^e^ Anti-retroviral therapy (ART), ^f^ Cotrimoxazole preventive therapy (CTX-P)
