# Supplementary material for: Impact of Anti-Retroviral Treatment and Cotrimoxazole Prophylaxis on Helminth Infections in HIV-Infected Patients in Lambaréné, Gabon
Source: PLoS Negl Trop Dis. 2015 May 20;9(5):e0003769. doi: 10.1371/journal.pntd.0003769 (PMC4439024; doi:10.1371/journal.pntd.0003769)
Supplement: S4 Table — (DOCX) [file pntd.0003769.s005.docx]

| **S4 Table 4. Time on ART for participants infected versus non-infected with one or more helminth infections, intestinal helminths or *Loa loa*.** | | | | |
| --- | --- | --- | --- | --- |
|  | **Data^a^ (n)** | **Not infected** | **Any infection** | **P-value^b^** |
| Time on ART^c^ in months (median, IQR^d^) | 130 | 21.5 (1.5-41.5) | 24.5 (4.5-44.5) | 0.21 |
|  |  | **No intestinal helminth infection** | **Intestinal helminth infection** |  |
| Time on ART^c^ in months (median, IQR^d^) | 130 | 25.0 (4.5-45.5) | 15.0 (3.0-34.0) | 0.56 |
|  |  | **No *Loa loa* infection** | ***Loa loa* infection** |  |
| Time on ART^c^ in months (median, IQR^d^) | 130 | 19.5 (0-39.0) | 34.5 (14.0-55.0) | 0.12 |
|  |  |  |  |  |

Median time on ART in months for patients who were diagnosed with one or more helminth infections versus those who had negative test results.

^a^ The first column shows for how many patients data were complete for each respective variable.

^b^ P-values were calculated using the Mann Whitney U as the distribution for time on ART was non-parametric.

^c^ Anti-retroviral therapy (ART), ^d^ Interquartile range (IQR)
